# Exploring the Blood Glucose-Lowering Potential of the Umami Peptides LADW and EEAEGT Derived from Tuna Skeletal Myosin: Perspectives from α-Glucosidase Inhibition and Starch Interaction

**DOI:** 10.3390/foods13020294

**Published:** 2024-01-17

**Authors:** Shuai Zhao, Shengbao Cai, Lixin Ding, Junjie Yi, Linyan Zhou, Zhijia Liu, Chuanqi Chu

**Affiliations:** 1 Faculty of Food Science and Engineering, Kunming University of Science and Technology, Kunming 650500, China; zhaoshuaikust1998@163.com (S.Z.); caikmust2013@163.com (S.C.); dlx2528@163.com (L.D.); junjieyi@kust.edu.cn (J.Y.); zhoulinyan916@hotmail.com (L.Z.); liuzhijia@kust.edu.cn (Z.L.); 2 Yunnan Engineering Research Center for Fruit & Vegetable Products, Kunming 650500, China; 3 International Green Food Processing Research and Development Center of Kunming City, Kunming 650500, China; 4 Yunnan International Joint Laboratory of Green Food Processing, Kunming 650500, China

**Keywords:** α-glucosidase inhibitory peptides, molecular docking, molecular dynamics simulation, weak interaction force, independent gradient model, starch hydrolysis inhibition

## Abstract

This study aimed to explore the potential of umami peptides for lowering blood glucose. Molecular docking results showed that the peptides LADW and EEAEGT bound to the active amino acid residues of α-glucosidase via hydrogen bonds and Van der Waals forces, a finding supported by an independent gradient model (IGM). Molecular dynamics (MD) simulations demonstrated that the peptides LADW and EEAEGT can decelerate the outward expansion of α-glucosidase and reduce amino acid fluctuations at the active site. In vitro findings indicated that the peptides LADW and EEAEGT showed potent inhibitory activity against α-glucosidase, with IC_50_ values of 4.40 ± 0.04 and 6.46 ± 0.22 mM, respectively. Furthermore, MD simulation and morphological observation results also revealed that LADW and EEAEGT alter starch structure and form weak interactions with starch through intermolecular hydrogen bonding, leading to the inhibition of starch hydrolysis. Peptides inhibit the ability of starch to produce reducing sugars after simulated gastrointestinal digestion, providing additional evidence of the inhibition of starch hydrolysis by the added peptides. Taken together, these findings suggest that consuming the umami peptides LADW and EEAEGT may alleviate postprandial blood glucose elevations via inhibiting α-glucosidase and starch hydrolysis.

## 1. Introduction

Diabetes is a prevalent chronic metabolic condition characterized by elevated blood glucose levels. As per the IDF Diabetes Atlas, in 2021, there were 537 million individuals aged 20–79 worldwide with diabetes, which is one of the leading causes of death in developed countries [1]. Furthermore, diabetes increases the risk of other diseases; studies indicate that COVID-19 patients with diabetes are at a higher mortality risk compared with the healthy population [2]. The primary focus in diabetes treatment is to lower and/or control blood glucose, particularly postprandial blood glucose levels. α-Glucosidase (EC:3.2.1.20) is considered one of the most vital enzymes in humans for carbohydrate hydrolysis. In clinical practice, acarbose is frequently used to inhibit α-glucosidase activity, thereby reducing the postprandial blood glucose increase. However, acarbose is associated with some adverse effects, including flatulence, diarrhea, and abdominal pain, among others [3]. This has led to the exploration of natural inhibitors as a safer and more dependable alternative to synthetic drugs. In addition, previous studies have indicated that modifying the starch structure to make it less digestible can effectively inhibit starch hydrolysis [4,5,6].

In our previous work, we identified four novel umami peptides (LADW, MEIDD, VAEQE, and EEAEGT) in the hydrolysate of tuna skeletal myosin through enzymatic hydrolysis with pepsin, trypsin, and chymotrypsin enzymes hydrolysis [7]. Umami peptides are regarded as a promising seasoning that improves food flavor and offers nutritional value [8]. From the perspective of food flavor, umami peptides are recognized for their strong umami taste characteristics [9]. Furthermore, previous studies have demonstrated that umami peptides may exhibit antioxidant activities and the ability to inhibit Angiotensin I-Converting Enzyme (ACE) and Dual Dipeptidyl Peptidase-IV (DPP-IV) [8,10]. It is important to emphasize that umami peptides must maintain their structures to exert their potential biological activity in the body. However, the digestive system contains numerous protein-digesting enzymes that can potentially destroy the structures of umami peptides. There is limited information available regarding the potential functional activities of umami peptides after passing through the gastrointestinal (GI) tract. This is likely because the resistance of umami peptides to GI tract digestion was not taken into account during the preparation and purification process. As a result, bioactivity may be lost after digestion by enzymes like pepsin, and other protein-digesting enzymes, which, as shown in previous studies, have a tendency to cleave hydrophobic amino acids, the primary constituents of umami peptides [10,11]. Furthermore, proline (Pro), methionine (Met), and alanine (Ala), as hydrophobic amino acids, are considered to be the amino acids mainly responsible for α-glucosidase inhibitory peptides (α-GIPs) [12]. Therefore, umami peptides designed with resistance to GI digestion may promise the potential to be effective α-GIPs. To date, the ability of peptides to alter starch structure and inhibit starch hydrolysis has been infrequently explored. Nevertheless, molecular dynamics (MD) simulation is considered a visual method for investigating the binding mechanism of starch and small-molecule compounds [5,6]. Additionally, scanning electron microscopy (SEM) has been employed to observe alterations in starch structure [13]. The umami peptides in this study were prepared through GI enzyme hydrolysis [7]. And it is worth noting that peptides prepared through GI enzyme hydrolysis are generally recognized for their resistance to digestion [14]. Based on this, this study explores whether the aforementioned umami peptides can function as peptides that lower blood glucose levels from the perspective of both their potential as α-GIPs and their impact on starch structure.

This work aims to investigate the potential of umami peptides to resist GI digestion and to lower blood glucose function through a combination of in silico and in vitro experiments. In this study, in silico analysis was used to preliminarily predict the potential activities and safety of four novel umami peptides, revealing their potential for lowering blood glucose levels. Using MD simulations and an independent gradient model (IGM), we conducted a comprehensive analysis of the potential mechanisms underlying umami peptide binding to α-glucosidase and investigated the effect of α-GIPs on starch structure. Additionally, the findings from computer simulations were validated through in vitro experiments. Ultimately, this study used molecular docking and MD simulation techniques to explain the mechanisms of peptide binding to α-glucosidase and starch, respectively. It obtained more precise results regarding peptides and receptor binding ability through IGM and morphological observations. The above methodology provides a more accurate way to elucidate the binding ability of peptides to receptors.

## 2. Materials and Methods

### 2.1. Material

α-Glucosidase from *Saccharomyces cerevisiae* (activity ≥ 30 U/mg) and porcine bile salt (purity ≥ 60.0%) were obtained from Yuanye Bio-Technology Co., Ltd. (Shanghai, China). Pepsin (activity ≥ 800 U/mg), pancreatin (activity ≥ 4000 U/g), and amyloglucosidase (activity ≥ 10,000 U/g) were obtained from Shanghai Ryon Biological Technology Co., Ltd. The p-nitrobenzene-α-D-glucopyranoside (pNPG, purity ≥ 99.0%) was purchased from Sigma-Aldrich (Shanghai, China). Peptides LADW and EEAEGT (purity ≥ 98.0%) were synthesized by ALL PEPTIDE Biology Co., Ltd. (Hangzhou, China). All other chemicals and solvents were of analytical grade.

### 2.2. Prediction of Potential Biological Activity and Physicochemical Properties

In the preliminary work, the potential biological activity and allergenicity of the four umami peptides were assessed by using the BIOPEP database [15] and AllerTOPv.2.0 [16], respectively. Additionally, their physicochemical properties were predicted by Innovagen (Innovagen AB, Lund, Sweden). The GI digestion tolerance of peptides was analyzed using BIOPEP database [15] and PeptideCutter (https://web.expasy.org/peptide_cutter/ (accessed on 20 July 2023)). The specific operation is as follows. Peptides were subjected to enzymatic hydrolysis using pepsin, trypsin, and chymotrypsin to obtain the preserved sequences.

### 2.3. Molecular Docking

The three-dimensional (3D) structure of the peptides LADW and EEAEGT were obtained by using PeptideConstructor (https://github.com/TinkerTools (accessed on 25 July 2023)). Subsequently, each peptide’s structure was optimized by using the general Amber force field (GAFF) of Avogadro [17]. Due to the unavailability of the 3D structure of α-glucosidase from *Saccharomyces cerevisiae*, we employed the crystal structure of isomaltase from *Saccharomyces cerevisiae* (PDB ID: 3A4A) as the template protein to construct the α-glucosidase structure using homology modeling methods. After checking the crystal structure and removing calcium ions that were not in the active site, the modeling procedure followed the method described by Jia et al. [18]. Before docking, non-polar hydrogens and Gasteiger charges were added to proteins and ligands by using AutoDockTools (ADT 1.5.7) [19]. In the semi-flexible docking mode employed, molecular docking was configured to treat a peptide as a flexible structure, while α-glucosidase protein was held in a rigid structure. Drawing upon previous investigations into the active amino acids and site of α-glucosidase [18,20,21,22], the location of the active docking box is delineated in Figure S1; the active docking box for α-glucosidase was defined with central coordinates (x = 25.38 Å, y = −2.75 Å, z = 18.19 Å) and dimensions of 52 × 68 × 62 Å. Molecular docking was performed by using Autodock Vina 1.1.2 software [23]. Molecular docking results were analyzed using PyMOL 2.5 (Open-source, Schrödinger, New York, NY, USA) and Discovery Studio Visualizer Client (Open-source, BIOVIA, San Diego, CA, USA).

### 2.4. ADMET Analysis

Peptides ADMET (absorption, distribution, metabolism, excretion, and toxicity) assessment was performed by using ADMETLab 2.0 [24], admetSAR @ LMMD [25], SwissADME [26], and the iDrug platform (https://drug.ai.tencent.com/console/cn/admet (accessed on 5 August 2023)). Results from each database were consolidated and analyzed to provide a comprehensive evaluation of the ADMET properties of the peptides.

### 2.5. MD Simulation

MD simulation was performed in the GROMACS 19.5 software package (https://manual.gromacs.org/ (accessed on 20 August 2023)) and utilized the Amber FF99SB-ILDN forcefield, which includes the GLYCAM_06j-1 forcefield. Two different systems, peptide-α-glucosidase and peptide-amylose, were constructed separately with different topology files.

#### 2.5.1. Peptide and α-Glucosidase Systems

The 3D structures of peptides and α-glucosidase were derived from the molecular docking results. Topology files for both the peptides and α-glucosidase were constructed by using the GAFF and Amber FF99SB-ILDN forcefield, respectively. First, the three-site transferable intermolecular potential (TIP3P) water model was incorporated into each complex system, and the NaCl concentration was set at 0.15 M. Second, energy optimization was performed by using the steepest-descent method, resulting in energy levels below 1000.0 kJ (mol^−1^ nm^−1^). Third, isothermal & isochoric simulation (NVT, 2 ns) and isothermal & isobaric simulation (NPT, 1 ns) were performed to ensure that the system performed MD simulations under the specified conditions of temperature (310.15 K) and pressure (1 bar). Ultimately, MD simulations were performed for 100 ns and accelerated by using a graphics processing unit processor.

#### 2.5.2. Peptide and Amylose Systems

The amylose model was constructed by connecting 26 α-D-glucose molecules through α-1,4-glycosidic bonds. The amylose model was constructed by using the AmberTools18 software package (https://ambermd.org/AmberTools.php (accessed on 20 August 2023)). The model for each peptide–amylose complex was constructed by using the PACKMOL program [27]. Topology files for peptides and amylose were constructed by using the GAFF and GLYCAM_06j-1 forcefields, respectively. MD simulations were performed following the procedures outlined in Section 2.5.1.

#### 2.5.3. Data Analysis of MD Simulation

After the MD simulation, snapshots of both the single amylose and peptide–amylose complex conformations were extracted at 20 ns intervals for analysis of amylose’s conformational changes. Moreover, the root mean square deviation (RMSD), radius of gyration (Rg), solvent accessible surface area (SASA), number of hydrogen bonds (H-bond number), root mean square fluctuation (RMSF), radial distribution functions (RDF), and energies were also extracted for further analysis in this study.

#### 2.5.4. Free Energy Landscape (FEL) and IGM Analyses

The FEL was obtained by using covariance matrix construction and principal component analysis (PCA), which leads to a clear observation of the Gibbs free energy in relation to structural stability [26]. Gibbs free energy was calculated by using the Boltzmann distribution. Following the method of Pan et al. [28], RMSD and Rg were extracted as two data columns. The Converting dot distribution to probability distribution v1.3 program was used to generate three columns, subsequently utilized for FEL plotting. Each peptide–α-glucosidase complex conformation with the lowest Gibbs free energy was examined for weak interaction force. The weak interaction force was assessed by using the IGM of the Multiwfn 3.7 program [29]. IGM is capable of detecting hydrogen bonds, van der Waals forces, and hydrophobic interactions between target fragments and other fragments. In this study, the analysis focused on weak interaction forces between each peptide and the surrounding residues within 5 Å. The computational approach for IGM followed the procedure outlined by Lu et al. [29]. Visual analysis was performed using VMD 1.9.3 software [30].

### 2.6. Inhibition Activity of α-Glucosidase

α-Glucosidase inhibitory activity was determined following the method of Zheng et al. [21], with slight modifications. Briefly, 80.0 μL of phosphate-buffered saline (PBS, pH = 6.8), 20.0 μL of α-glucosidase (7 U/mL, dissolved in PBS), and 20.0 μL of the peptide (dissolved in PBS) were mixed and incubated at 37 °C for 15 min. Subsequently, 20.0 μL of pNPG (2.5 mM, dissolved in PBS) was added to the mixture, followed by another incubation at 37 °C for 15 min. To terminate the reaction, 60 μL of Na_2_CO_3_ (0.2 M, dissolved in PBS) was added, and the absorbance was measured at 405 nm using a SpectraMax M5 microplate reader (Molecular Devices, Sunnyvale, CA, USA). The α-glucosidase inhibitory rate (%) was determined using the following formula:

α-glucosidase inhibitory rate (%) = [1 − (A_sample_ − A_sample blank_)/(A_control_ − A_control blank_)] × 100

where A_sample_ and A_sample blank_ were the absorbance values with and without α-glucosidase added, respectively; A_control_ and A_control blank_ were the absorbance values with and without peptide solution added, respectively, after replacing α-glucosidase solution with PBS solution. The above solution without addition was replaced by PBS solution.

### 2.7. Starch–Peptide Interactions

#### 2.7.1. Preparation of Sample

The starch–peptide complex was prepared with slight modifications following the method outlined by Lu et al. [4]. A 5% (*w*/*v*) starch suspension was prepared by weighing 0.5 g of soluble starch and adding distilled water. Subsequently, peptide (0.1%, *w*/*v*) was added to the starch suspension, and the mixture was stirred in boiling water for 30 min to obtain the starch–peptide complex solution. After heating, the starch–peptide complexes were equilibrated at 37 °C for 30 min. The samples were then rapidly frozen in liquid nitrogen and subsequently freeze-dried for 48 h using a vacuum freeze dryer (Alpha 1-2 LD plus, Christ, Germany), after rapid freezing in liquid nitrogen. The control group consisted of individually prepared starch.

#### 2.7.2. In Vitro Simulation Digestion of Starch and Starch–Peptide

In vitro simulated digestion was conducted following the method described by Xia et al. [31], with slight modifications. Electrolytes in simulated gastric fluid (SGF) and simulated intestinal juice (SIF) were configured according to Table S1. A quantity of 0.1 g of starch and starch–peptide complex were added to 750.0 μL of SGF. Then, calcium chloride (0.5 µL, 0.3 M) and porcine pepsin (200.0 µL, 12,500 U/mL) were added, and the pH was adjusted to 2.0 ± 0.2 by using 1.0 M HCl. After each sample weight was made up to 2.0 g, the mixture was incubated in the dark on a shaker (200 rpm, 37 °C) for 2 h. After SGF digestion, SIF (1.1 mL), calcium chloride (0.4 µL, 0.3 M), bile salts (20.0 µL, 10.0 mM), and pancreatin (200.0 µL, 500 U/mL) were added to gastric juice samples, and the pH was adjusted to 7.5 ± 0.2 using 1.0 M NaOH. After each sample weight was made up to 4.0 g, the mixture incubated in the dark on a shaker (200 rpm, 37 °C) for 2 h. After SIF digestion, 0.5 mL of digestive juices was extracted from each sample, dissolved in 2.0 mL of ethanol (95%, *v*/*v*), and stored at −20 °C [32].

#### 2.7.3. Determination of Reducing Sugars

The 2-hydroxy-3,5-dinitrobenzoic acid (DNS) method was used for the determination of reducing sugars, with slight modifications, following the procedure outlined by Goh et al. [32]. DNS was prepared according to Table S2. After centrifugation (2500× *g*, 10 min) of the 2.5 mL digestive juice–ethanol solution, 50 μL of the supernatant was placed in a glass test tube. Additionally, the same volume of glucose standard solution (10 mg/mL) and deionized water were used as control and blank groups, respectively. A 1% amyloglucosidase solution (*w*/*v*) was prepared in acetate buffer (0.1 M, pH = 5.2), and then 0.25 mL of this solution was added to the sample and allowed to stand at 25 °C for 30 min. After completion, 0.75 mL of DNS mixture (DNS: 4M NaOH: 0.5 mg/mL glucose standard solution, 4:1:1, *v*/*v*/*v*) was added, followed by heating in boiling water for 15 min and subsequent cooling to room temperature. After cooling, deionized water was added for dilution, and the absorbance values were measured at 530 nm using a SpectraMax M5 microplate reader (Molecular Devices, Sunnyvale, CA, USA). In addition, the accuracy of the method was verified by using a glucose standard solution ranging from 2.5–40 mg/mL. The reducing sugar content (mg/g) was calculated using the following formula:

Reducing sugar content (mg/g) = (A_sample_ − A_blank_)/(A_control_ − A_blank_) × glucose standard solution concentration × dilution factor × volume of digestive juices × 1/sample weight total

where A_sample_, A_blank_, and A_control_ were the absorbance values of the sample, glucose standard solution (10 mg/mL), and deionized water, respectively. The dilution factor was considered to be 5.

Inhibition rate of reducing sugar production (%) = (1 − C_complex_/C_starch_) × 100

where C_complex_ and C_starch_ were the reducing sugar content of starch–peptide complex and starch, respectively.

#### 2.7.4. Starch Granule Morphological Observation

A small quantity of starch and starch–peptide complex samples were secured onto the sample stage using conductive glue. The morphology of the samples after gold spraying was observed using SEM (VEGA3 TESCAN, Brno, Czech Republic) with a voltage of 20.0 kV.

### 2.8. Statistical Analysis

In vitro experiment results were expressed as mean ± standard deviation (S.D.) (*n* = 3). Tukey’s test with one-way ANOVA was applied to detect significant differences (*p* < 0.05) by using Origin 8.5 (Northampton, MA, USA).

## 3. Results and Discussion

### 3.1. Potential Activity Analysis and Physicochemical Properties of Peptides

In the preliminary work, we identified four umami peptides and evaluated their potential biological activities using the BIOPEP-UWM database [15]. Among these umami peptides, LADW and EEAEGT demonstrated potential inhibitory activity against ACE, DPP-IV, and α-glucosidase (Table 1). Additionally, VAEQE and MEIDD exhibited potential inhibitory activity against DPP-IV (Table 1). Furthermore, AllerTOPv.2.0 [16] was used to predict allergenicity, revealing that the peptides LADW and EEAEGT may not be allergenic, while the peptides VAEQE and MEIDD could be allergenic (Table 1). α-Glucosidase inhibitors, such as acarbose, are usually administered orally and work by inhibiting intestinal α-glucosidase to reduce the digestion and absorption of carbohydrates. Likewise, umami peptides enter the body through the mouth, where they regulate food flavor, and then proceed into the GI tract for digestion. Our previous study demonstrated that the prepared umami peptides resisted hydrolysis in the GI tract [7]. In addition, through GI enzymatic hydrolysis of the peptides, we found that the peptides EEAEGT and LADW have certain resistance to GI digestion (Table S3). Consequently, peptides LADW and EEAEGT were selected for further investigation as potential α-GIPs. Sensory evaluation and predicted isoelectric point (pI) values of the umami peptides indicated their high-water solubility. Furthermore, adequate water solubility was essential for peptides to exert their functional activity upon entering the human body [21].

### 3.2. Molecular Docking

**Figure 1:**
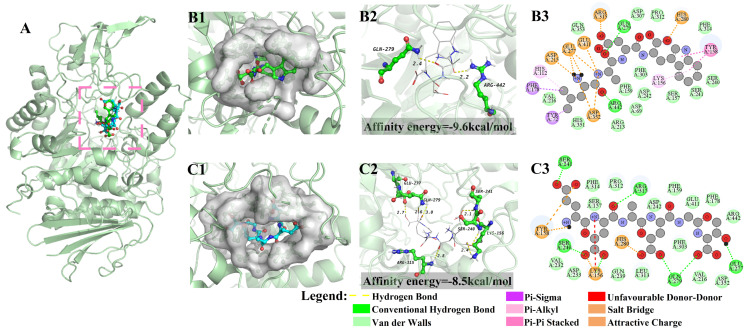
Molecular docking results of peptides LADW and EEAEGT binding α-glucosidases. Panel (**A**) displays the binding sites of peptide and α-glucosidases. Panels (**B**,**C**) show the results of peptides LADW and EEAEGT binding to α-glucosidases, respectively. The numbers 1 to 3 respectively represent the 5 Å amino acid residues around the peptide, the hydrogen bond interaction of three-dimensional images, and the intermolecular interaction binding two-dimensional images of peptide and α-glucosidases.

Molecular docking studies are frequently performed for potential drug screening. Additionally, numerous studies involving food-derived bioactive peptides have also been screened and validated through molecular docking [7,21]. Figure 1 displays the results of the molecular docking involving the peptides LADW and EEAEGT with α-glucosidase. The present study observed that the peptides LADW (green) and EEAEGT (blue) were situated within the active pocket of α-glucosidase, as presented in Figure 1(B1,C1). Figure 1(B2) shows that peptide LADW formed two hydrogen bonds with the amino acid residues Gln 279 and Arg 442 in α-glucosidase, with an average bond distance of 2.30 Å. Figure 1(C2) demonstrates that the peptide EEAEGT formed seven hydrogen bonds with amino acid residues Lys156, Ser240, Ser241, Glu277, Gln279, and Arg315 in α-glucosidase, with an average bond distance of 2.59 Å. Meanwhile, acarbose and the positive control peptide also formed a stable intermolecular interaction force with the aforementioned amino acid residues of α-glucosidase (Figure S2). The present study showed that the amino acid residue Gln 279 in α-glucosidase formed hydrogen bonds with the peptides LADW and EEAEGT. Previous studies also regarded Gln 279 as the primary amino acid residue of α-glucosidase involved in ligand binding [20,22]. Meanwhile, the peptides LADW (Figure 1(B3)) and EEAEGT (Figure 1(C3)) were combined with α-glucosidase by amino acid residues to form van der Waals forces, summarized in Table S4. However, there was no obvious difference in the number of van der Waals forces formed when the peptides LADW and EEAEGT bind to the α-glucosidase. Furthermore, the result indicated that alkyl interactions were considered to be unique interaction forces between the peptide LADW and α-glucosidase (Table S4). Furthermore, considering that smaller affinity can indicate potentially better binding ability [20], from the perspective of affinity energy, the binding capacity of these peptides with α-glucosidase ranked as LADW > EEAEGT, as shown in Table S4.

To improve the reliability of screened bioactive peptides, depending solely on molecular docking studies and database predictions may not be sufficient. Therefore, in addition to these methods, the present study used MD simulations and in vitro experiments to further investigate the effect of α-GIPs. Prior to assessing their activity, the peptides LADW and EEAEGT were first analyzed by ADMET in the present study.

### 3.3. ADMET Analysis

The intestinal stability, non-toxicity, and absorbability of peptides were considered important indicators for identifying bioactive peptides. In our previous work, these peptides were also confirmed to be non-toxic by sensory evaluation, and the peptides LADW and EEAEGT were obtained by hydrolysis with three common enzymes (pepsin, trypsin, and chymotrypsin) in the GI tract [7]. Furthermore, in silico GI digestion of the peptides result confirmed that both peptides have certain resistance to GI digestion. Therefore, the potential α-GIPs identified in this study were presumed to withstand hydrolysis and exhibit non-toxicity. In addition to the previous exploration, this study conducted ADMET analysis on the peptides LADW and EEAEGT using four database predictions, as presented in Table 2. In absorption analysis, human intestinal absorption (HIA) was deemed the pivotal pharmacokinetic attribute for delivering a compound’s biological activity [33]. Additionally, Caco-2 and Log P were used to represent human intestinal penetration and water solubility, respectively [24]. HIA results for peptides vary among the four databases primarily due to differences in data collection timing and prediction algorithms. Consequently, this study favors the results from emerging databases like ADMETLab 2.0 [24] and the iDrug platform. It suggests that the peptides LADW and EEAEGT exhibit robust HIA ability. Furthermore, α-glucosidase inhibition typically operates in the intestinal tract, necessitating high HIA capacity as a prerequisite for the potential functions of the peptides LADW and EEAEGT. The peptides LADW and EEAEGT exhibited low human intestinal penetration but high water solubility. However, there was no marked correlation between α-glucosidase inhibition and human intestinal permeability, potentially limiting the application of peptides in other functions. In addition, the favorable water solubility findings aligned with the physicochemical property analysis.

In metabolism analysis, six CYP450 enzymes (CYP1A2, 2C9, 2C19, 2D6, 3A4, and 3A5) metabolize more than 90% of drugs [34]. The degree of CYP450 inhibition is positively correlated with drug metabolism [35]. In this study, we investigated the inhibitory effect of the peptides LADW and EEAEGT on CYP450 enzymes (1A2, 2C19, 2C9, 2D6, and 3A4). The results indicate that these peptides did not obviously inhibit CYP450 activity (Table 2), suggesting that they are unlikely to affect CYP450-mediated drug metabolism.

Regarding distribution and excretion, the results in Table 2 indicate that the peptides LADW and EEAEGT are unlikely to penetrate the blood–brain barrier (BBB) and exhibit only minor binding to plasma proteins (PP). However, they can be metabolized and eliminated by the human body. Consequently, this study suggests that the potential α-GIPs can exert their effect and undergo excretion. However, their limited ability to cross the BBB and weak binding to PP may restrict their utility in other applications. Furthermore, the peptides LADW and EEAEGT were predicted to be non-toxic in terms of carcinogenicity, rat oral acute toxicity, drug-induced liver injury (DILI), and Ames toxicity (Table 2).

In summary, ADMET analysis indicated that the peptides LADW and EEAEGT were efficiently absorbed, metabolized, and excreted in the human body, suggesting their potential as bioactive peptides. Therefore, we further performed additional MD simulations and in vitro experiments to confirm the potential of the peptides LADW and EEAEGT as α-GIPs and starch hydrolysis inhibitors.

### 3.4. MD Simulations

#### 3.4.1. Peptide-α-Glucosidase Complex Systems

**Figure 2:**
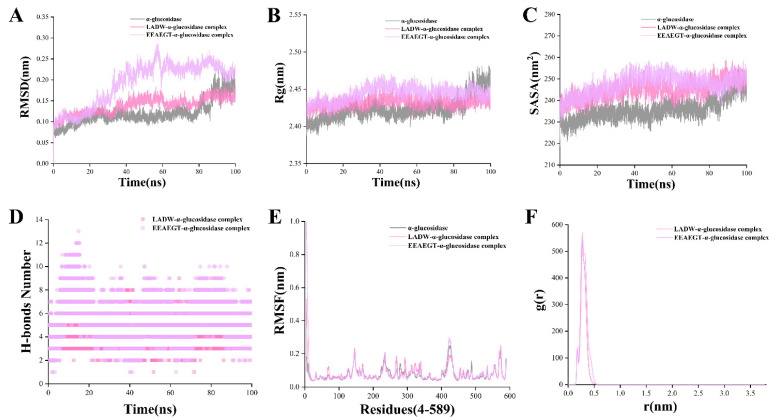
Molecular dynamics (MD) simulation results of peptide-α-glucosidase complexes and single α-glucosidases. Panels (**A**–**F**) respectively represent the root mean square deviation (RMSD), radius of gyration (Rg), solvent accessible surface area (SASA), number of hydrogen bonds (number of H-bonds), root mean square fluctuation (RMSF), radial distribution functions (RDF) for peptide-α-glucosidase complexes.

Figure 2 illustrates the results of a 100 ns MD simulation for a peptide-α-glucosidase complex/single α-glucosidase. The RMSD value is a critical indicator for assessing the conformational stability of the protein/complex, where relatively stable RMSD values signify higher conformational stability [20]. The results indicated that the RMSD values for the LADW- and EEAEGT-α-glucosidase complex were stable at 35 and 40 ns, respectively. Notably, the RMSD value following stabilization of the LADW-α-glucosidase complex was lower than that of the EEAEGT-α-glucosidase complex, as shown in Figure 2A. In contrast, the RMSD value of the single α-glucosidase sharply increased at 85 ns and subsequently stabilized. Furthermore, the RMSD value of the positive control peptide exhibited stabilization after 65 ns (Figure S3A). Based on these findings, this study concludes that the conformation of the LADW-α-glucosidase complex was the most stable, followed by the EEAEGT-α-glucosidase complex, while the single α-glucosidase was found to be unstable. Therefore, it is inferred that the potential binding ability between peptide LADW and α-glucosidase was superior to that of peptide EEAEGT.

The Rg value is closely associated with the tightness of the protein/complex [20]. The study observed a slight increase in the Rg values for the peptide LADW- and EEAEGT-α-glucosidase complexes, both showing an increase of approximately 0.02 nm (Figure 2B). Manrya et al. also observed an increase in the Rg value of the ligand-α-glucosidase complex after MD simulations [36], supporting the reliability of the MD simulation results in this study. Moreover, the Rg for the positive control peptide demonstrated an increase compared to the 0 ns reference point (Figure S3B). Interestingly, similarly to the observed change in the RMSD value, the Rg value of the single α-glucosidase experiences a sudden increase at 85 ns, indicating instability at its active site and potential susceptibility to carbohydrate binding, leading to monosaccharide production. Additionally, Rg analysis indicates that during 100 ns MD simulations, peptides LADW and EEAEGT are likely to be closely enveloped by the amino acid residues at the α-glucosidase active site, potentially enhancing the stability of α-glucosidase and inhibiting its activity. The SASA value, as shown in Figure 2C, revealed an obvious increase in the SASA value of the complex/protein. SASA serves as an indicator predicting the degree of the conformational changes during the binding process, which is generally in line with changes in Rg [37]. The fluctuation range of both the LADW-α-glucosidase and EEAEGT-α-glucosidase complexes was found to be similar, fluctuating within the range of 240–250 nm^2^. In contrast, the single α-glucosidase exhibited fluctuations within the range of 230–245 nm^2^. For the positive control peptide, the fluctuation in the SASA value closely parallels that observed in the peptide under investigation (Figure S3C). By combining Rg analysis, this study indicated that, firstly, the SASA changes in the three different systems were consistent with the Rg results, confirming the precision of the MD simulation. Secondly, the increased SASA fluctuations values indicated that the conformation of α-glucosidase became more stable upon peptide binding.

In Figure 2D, during 100 ns MD simulations, hydrogen bonds consistently formed between the peptides LADW or EEAEGT and α-glucosidase. Simultaneously, the hydrogen bonds between the peptide and α-glucosidase persist consistently in the positive control peptide (Figure S3D). Molecular docking studies have highlighted the significance of hydrogen bonds as key intermolecular interactions. Results indicated that the number of hydrogen bonds between peptides LADW or EEAEGT and α-glucosidase remained constant during the MD simulations, with no obvious difference in the number of hydrogen bonds between them. Furthermore, it was observed that the MW and active group of the peptide LADW were lower than those of the peptide EEAEGT (Table 1). Compounds with higher MW and more active groups may form more hydrogen bonds with active amino acids in proteins. Consequently, this study hypothesized that, in terms of forming hydrogen bonds, the peptide LADW exhibited obviously stronger binding capacity to α-glucosidase compared with the peptide EEAEGT. RMSF is an important indicator for assessing the stability of amino acid residues within protein [20], helping deduce alterations in active amino acid residues. In the presence of the peptides LADW and EEAEGT, the RMSF values of the active amino acid residues in α-glucosidase were slightly lower than those of single α-glucosidase, specifically at amino acid residue numbers 214–241 and 273–290. The positive control peptide demonstrated a dampening effect on the fluctuations in the RMSF values of the aforementioned amino acids (Figure S3E). Notably, the RMSF value of amino acid residue Gln279 in both complexes, which form hydrogen bonds with both peptides LADW (0.0562) and EEAEGT (0.0598), was obviously lower than that of single α-glucosidase (0.1426) (Figure 2E). Based on the results, it can be observed that in the presence of peptides, the fluctuations in active amino acid residues were notably inhibited compared with those in single α-glucosidase, implying that the peptides LADW and EEAEGT exhibited inhibitory effects on α-glucosidase. RDF can characterize the distance relationship between a ligand and a receptor [37]. In this study, we analyzed the RDF of the ligand to the protein surface to obtain the average density during the stable period of MD simulation, as depicted in Figure 2F. The RDF results showed that the peptides LADW and EEAEGT exhibited a maximum peak at 0.25–0.3 nm, signifying that both peptides formed short-distance and strong binding hydrogen bonds with α-glucosidase. Moreover, the peak heights of LADW-α-glucosidase and EEAEGT-α-glucosidase were similar, suggesting that the average distribution density of both peptides was comparable, which was consistent with the observed changes in hydrogen bond numbers (Figure 2D). Furthermore, the RDF maximum peak for the positive control peptide was observed at 0.3–0.35 nm, suggesting that the binding hydrogen bonds between the positive control peptide and α-glucosidase were weaker compared with those of the peptides in this study (Figure S3F).

Additionally, we extracted the Lennard–Jone (LJ) and Coulomb interaction (Coul) between each peptide and α-glucosidase, calculating the total energy (Table S5). Generally, the total energy value is used to assess the strength of the interaction between a ligand and a receptor, with a larger absolute value indicating a stronger and more stable interaction [21,37]. The results showed that the absolute total energy value of the peptide LADW-α-glucosidase complex was slightly higher than that of the peptide EEAEGT-α-glucosidase complex, indicating that the peptide LADW may have a stronger binding ability to α-glucosidase compared with the peptide EEAEGT.

**Figure 3:**
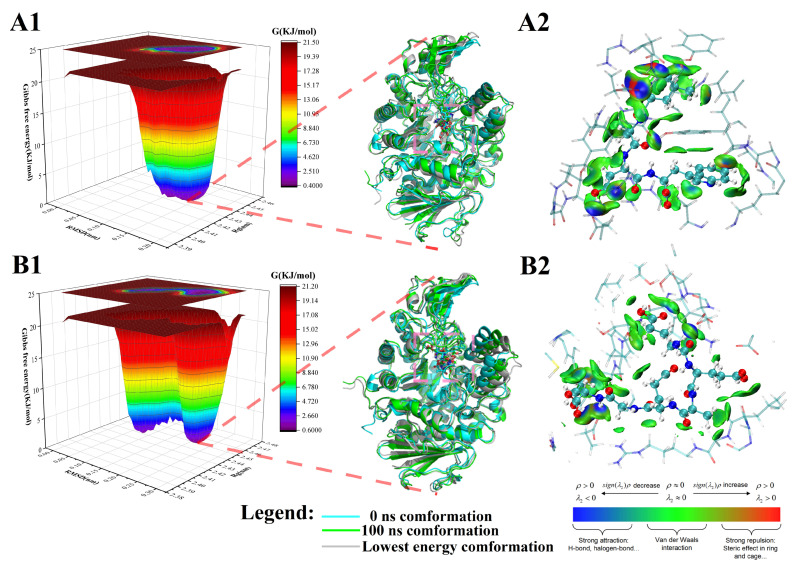
The free energy landscapes (FEL), three different conformation comparisons, and the weak interaction of peptides and α-glucosidases. Panels (**A**,**B**) show the results of peptides LADW and EEAEGT binding to α-glucosidases, respectively. The number 1 represents the FEL and the initial (cyan), lowest energy (grey), and final (green) comparison. The number 2 represents weak interaction between peptides and surrounding residues within 5 Å of α-glucosidases.

In addition, Figure 3 presents the FEL maps obtained from RMSD and Rg analyses. We extracted the initial conformation (0 ns), lowest energy conformation, and final conformation (100 ns) from MD simulations. Furthermore, in the lowest-energy conformation, we analyzed the weak interaction forces between the peptide and the surrounding residues within a 5 Å using IGM. This study observed that the peptides LADW and EEAEGT formed stable binding interactions within the active site of α-glucosidase. Their conformation positional changes during the initial and final stages of the MD simulations were minimal, as depicted in Figure 3(A1,B1). During the LADW- and EEAEGT-α-glucosidase complexes of 0 ns and 100 ns, the conformation of α-glucosidase exhibited a slight outward expansion, which was also supported by findings from Rg and SASA analyses. Furthermore, throughout the MD simulation, the conformation of the α-glucosidase in the positive control peptide-α-glucosidase complex also exhibited a slight expansion outward (Figure S4(A1,B1)). The IGM analysis of the lowest-energy conformation revealed an obvious presence of van der Waals forces and a limited number of hydrogen bond interactions between the peptides LADW (Figure 3(A2)) and EEAEGT (Figure 3(B2)) and the surrounding residues within 5 Å. These findings were consistent with the results of the intermolecular force analysis obtained from molecular docking. Simultaneously, the primary interaction forces between the positive control peptide and α-glucosidase were the van der Waals force and hydrogen bonding (Figure S4(A2,B2)). This observation underscores that the weak interaction force established between the peptide and α-glucosidase was predominantly attributed to van der Waals forces and hydrogen bonding. Interestingly, the present study indicated that the peptide LADW can form a greater number of hydrogen bonds with surrounding residues within 5 Å by IGM analysis. This finding supports the notion that the binding ability of the peptide LADW to α-glucosidase was stronger than that of the peptide EEAEGT, implying that the peptide LADW exhibits superior inhibitory effect. MD simulations analyses suggested that the peptides LADW and EEAEGT occupied the active site of α-glucosidase and interacted with active amino acid residues, resulting in reduced fluctuations in active amino acids and stabilizing the conformation of α-glucosidase. When comparing their abilities to bind to α-glucosidase, we found that the peptide LADW was obviously stronger than the peptide EEAEGT. However, it is essential to validate this result through in vitro experiments.

#### 3.4.2. Peptide–Amylose Complex Systems

**Figure 4:**
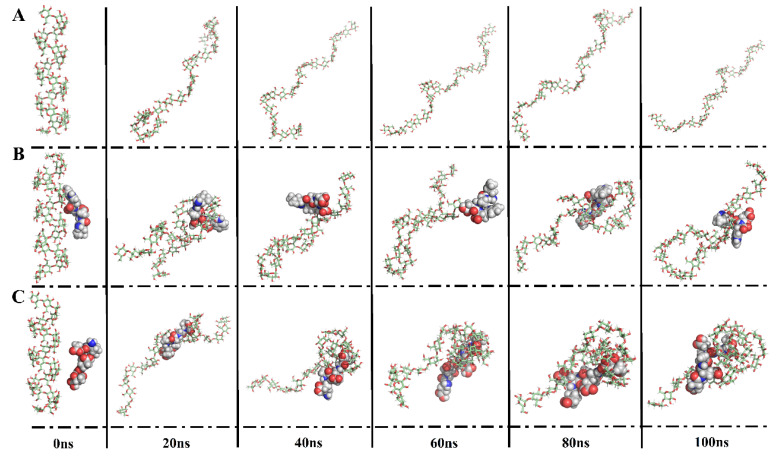
Conformational transitions every 20 ns of single amylose (**A**), LADW–amylose complex (**B**), and EEAEGT–amylose complex (**C**) in 100 ns MD simulation.

**Figure 5:**
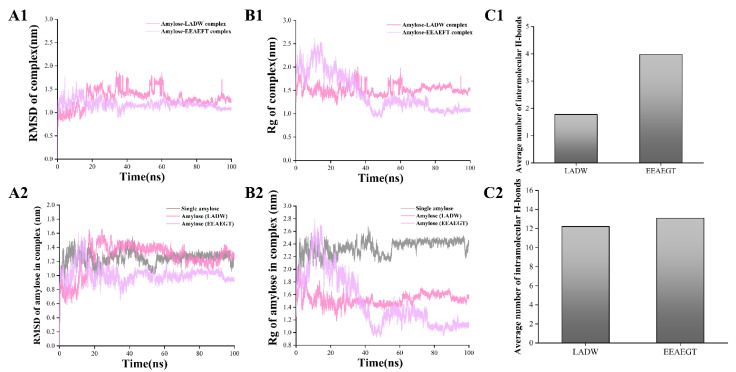
MD simulation results of peptide–amylose complex and single amylose. Panels (**A**–**C**) respectively represent the RMSD, Rg, and average number of hydrogen bonds (intermolecular and intramolecular) for the peptide–amylose complex. In panels (**A**,**B**), the numbers 1 and 2 respectively represent peptide–amylose complex and amylose in complex.

To investigate the interaction between peptides and starch, the present study performed 100 ns MD simulations for each peptide with amylose. In the initial conformation, each peptide was individually placed around amylose, followed by MD simulations. In Figure 4, during the 100 ns MD simulations, it can be observed that amylose coils around the peptides LADW and EEAEGT, especially around EEAEGT. Moreover, a single amylose chain extended continuously with the helix structure gradually disappearing, consistent with previous findings [6]. The peptides LADW and EEAEGT individually bound to amylose, forming intermolecular hydrogen bonds. Moreover, the average number of intermolecular hydrogen bonds for the peptide EEAEGT was obviously higher than that of peptide LADW (Figure 5(C1)). The wrapping of amylose around the peptides demonstrated that the peptides LADW and EEAEGT formed intermolecular hydrogen bonds with amylose, as shown in Figure 4B,C. In the presence of different peptides, the intramolecular hydrogen bonds of amylose exhibited slight alterations (Figure 5(C2)). Additionally, the findings indicated that the average number of intramolecular hydrogen bonds for the peptide EEAEGT was slightly higher than that of the peptide LADW. However, the increased compactness of amylose aggregation in the presence of the peptide EEAEGT may contribute to the enhanced intramolecular interaction force of amylose. Zhu et al., found that polyphenols with higher MW or more hydrogen bond donors exhibited stronger binding to amylose through MD simulations [5]. Likewise, the MW or hydrogen bond donors of the peptide EEAEGT were obviously higher than those of the peptide LADW. Furthermore, the number of hydrogen bonds indicated that the binding ability of the peptide EEAEGT to amylose was also stronger than that of the peptide LADW.

RMSD is a crucial indicator for characterizing complex stability; relatively stable RMSD values signify enhanced conformational stability [20]. In Figure 5(A1), the RMSD values of the peptides LADW and EEAEGT–amylose complexes reached stability at 65 and 40 ns, respectively. In the presence of peptides, the RMSD values in amylose changes were comparable to those in peptide–amylose complexes, while RMSD values of single amylose reached equilibrium after 55 ns (Figure 5(A2)). Additionally, the conformational alterations in the extracted amylose paralleled the RMSD analysis findings (Figure 4). Inhibiting amylose hydrolysis may be more effective when the RMSD value stabilizes earlier. Consequently, the peptide EEAEGT exhibited obviously greater amylose hydrolysis inhibition compared with LADW. The Rg was considered to be closely associated with the degree of tightness of the amylose itself [6]. As shown in Figure 5(B1), the Rg values of the peptide LADW- and EEAEGT-amylose complexes stabilized at 65 and 50 ns, respectively. In the presence of peptides, the Rg values of amylose exhibited a similar trend to those of the peptide LADW- and EEAEGT-amylose complexes, while the Rg values of single amylose molecules stabilized after 55 ns (Figure 5(B2)). Based on the conformational analysis in Figure 4, it is clear that the Rg value reflects the compactness of amylose under three different conditions, with more tightly packed amylose structures having lower Rg values. After analyzing the conformation and Rg of amylose, it was found that the presence of the peptides LADW and EEAEGT resulted in a more compact amylose structure, with EEAEGT having a marked effect. Additionally, the conformational analysis revealed that amylose exhibited a coiled structure, leading to the compact state depicted in Figure 4. This suggests that under the present conditions, amylose was less prone to hydrolysis. Furthermore, the findings indicated that the peptide EEAEGT had a stronger ability to modify the amylose conformation in comparison with the peptide LADW. Consequently, it is probable that the peptide EEAEGT may exhibit a stronger ability to inhibit amylose hydrolysis.

In addition to these, the total energies of the peptide–amylose complexes were calculated as −138.86 ± 68.14 for LADW and −176.53 ± 100.75 for EEAEGT (Table S5), suggesting that both peptides exhibit a strong binding ability to amylose, with EEAEGT potentially having a stronger binding ability than LADW. Taken together, when compared to single amylose, the α-1,4-glycosidic linkages within the peptide–amylose complexes could be concealed due to tight coiling. Moreover, intramolecular and intermolecular interaction forces may enhance the stability of α-1,4-glycosidic linkages, consequently decreasing the hydrolysis of amylose into monosaccharide. Furthermore, multiple indicators provide evidence that the peptide EEAEGT possesses a stronger capability to inhibit amylose hydrolysis compared with LADW. However, this result requires validation through in vitro experiments.

### 3.5. In Vitro Experimental Verification

#### 3.5.1. Inhibition Activity of α-Glucosidase

**Figure 6:**
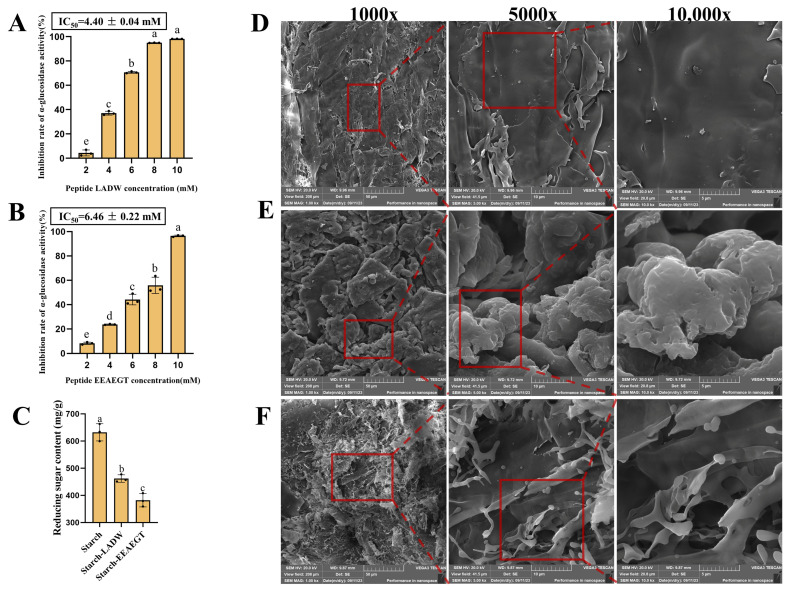
Inhibition ability of two peptides respectively towards α-glucosidases and starch hydrolysis. Panels (**A**,**B**) respectively represent inhibition rates of α-glucosidases by peptides LADW and EEAEGT of different concentrations. Panel (**C**) represents the reducing sugar content of starch and peptide–starch complex after simulated gastrointestinal digestion. Panels (**D**–**F**) respectively represent scanning electron microscope (SEM) pictures of starch, LADW-starch complex, and EEAEGT-starch complex. All the values are expressed as means ± SD (*n* = 3); the different letters indicate significant differences (*p* < 0.05).

In vitro experiments were employed as one of the methods to assess and validate the findings of the in silico simulations [20,21]. Figure 6A,B illustrate the inhibitory ability of peptides LADW and EEAEGT against α-glucosidase. Both peptides displayed a dose-dependent increase in their inhibitory activity with rising peptide concentration. At a concentration of 10 mM, the peptides LADW and EEAEGT displayed inhibitory rates of 98.15% and 96.44%, respectively. Figure 6A,B present the IC_50_ values for the peptides LADW and EEAEGT, indicating the concentration needed for 50% inhibition. The peptide LADW demonstrated the strongest inhibitory activity, with an IC_50_ value of 4.40 ± 0.04 mM, significantly lower than the IC_50_ value of EEAEGT (6.46 ± 0.22 mM) (*p* < 0.05), indicating that the peptide LADW possesses significantly stronger α-glucosidase inhibitory activity compared with EEAEGT. Notably, these findings were consistent with the outcomes of molecular docking, MD simulation, and IGM analysis. Furthermore, the α-GIPs exhibited lower IC_50_ values compared with those reported for peptides of the same chain length in previous studies [38,39,40]. This underscored the strong α-glucosidase inhibitory activity of the peptides LADW and EEAEGT.

#### 3.5.2. Reducing Sugar Content and Morphological Analysis of the Sample

After GI digestion, a portion of the starch was hydrolyzed by pancreatin, producing monosaccharides that can elevate blood glucose levels in the human body. Hence, this study assessed the peptides’ potential to inhibit starch hydrolysis by measuring the reducing sugar content of starch and peptide–starch complexes following digestion. Before analyzing the reduced sugar content of the samples, we employed multiple concentrations of a glucose standard solution to validate the accuracy of our method. A linear curve and an R^2^ value of 0.99712 were obtained (Figure S5). Figure 6C depicts the resulting reducing sugar content from starch hydrolysis, both with and without peptides, following GI digestion. The results indicate a significant reduction in starch hydrolysis in the presence of the peptides (*p* < 0.05). Notably, the peptide EEAEGT (inhibition rate of reducing sugar production: 26.87 ± 1.50%) demonstrated a significantly stronger capacity to inhibit starch hydrolysis compared with that of LADW (39.56 ± 0.82%) (*p* < 0.05). Goh et al. found that bread enriched with catechins yielded significantly lower (40%) reduced sugar content following GI digestion [32], consistent with the findings of this study. Furthermore, the effect of peptides on starch structure also was investigated in this study by using SEM.

Furthermore, this study investigated the impact of peptides on starch structure by using SEM. Starch presents a continuous and relatively smooth surface morphology (Figure 6D). However, the addition of peptides induces a transformation in starch, resulting in an irregular and rougher appearance (Figure 6E,F). This alteration may be attributed to the peptides disrupting the hydrogen bonds between starch molecules, resulting in a fragmented structure and the formation of lumps and roughness. He et al. previously reported similar effects, indicating that the interaction between quercetin and starch heightened the surface roughness of starch and disrupted the hydrogen bonds within the starch [13]. Under the SEM magnification of 1000×, in comparison to the LADW-starch complex, EEAEGT caused the starch to form a network-like surface structure and decreased starch continuity. This suggests a potentially stronger capability to disrupt the internal hydrogen bonds of starch (Figure 6E,F). Additionally, MD simulation results support these observations, showing that both peptides can form hydrogen bonds with amylose, resulting in amylose coiling around the peptides. This suggests that peptides could potentially disrupt the connect bonds between starch molecules. Consequently, the morphological observations are consistent with the results of MD simulations. Furthermore, this conclusion receives further support from the assessment of reducing sugar content. Integrating computer simulations with starch hydrolysis inhibition and morphological observation, the order of effectiveness in altering starch among these peptides was EEAEGT > LADW.

Although computer simulation and in vitro experimental findings confirmed the substantial inhibitory efficacy of the peptides LADW and EEAEGT on α-glucosidase and starch hydrolysis, but they could not fully simulate the actual situation in the human body, especially in the gut. Computer simulation and in vitro experimental findings lack in vivo experiments, and further studies are needed to substantiate them. At the same time, it is still possible that the methods give a practically useful approach to rapidly selecting peptides with inhibitory effects on α-glucosidase and starch hydrolysis.

## 4. Conclusions

This study aimed to investigate the potential functional activities of four umami peptides derived from tuna skeletal myosin. In silico modeling suggested that the peptides LADW and EEAEGT were potential α-GIPs, and AEDMT bioinformatic studies indicated they had bioactive properties. Results from molecular docking indicated that the hydrogen bonds and van der Waals forces were the main interaction forces between α-GIPs and the active amino acid residues of α-glucosidase. Moreover, MD simulations demonstrated that the peptides LADW and EEAEGT can decelerate the outward expansion of α-glucosidase and reduce amino acid fluctuations at the active site. In the conformations extracted by the FEL map, peptides were always bound to the active site of α-glucosidase via hydrogen bonds and van der Waals forces through IGM analysis. In vitro experiments confirmed that the peptides LADW (IC_50_ value: 4.40 ± 0.04 mM) and EEAEGT (IC_50_ value: 6.26 ± 0.22 mM) exhibit α-glucosidase inhibitory activity. The α-glucosidase inhibitory activity of these peptides ranked in the order LADW > EEAEGT, which was consistent with the results of computer simulation. Additionally, our study, through computer simulation and in vitro experiments, found that α-GIPs could alter the starch structure. MD simulations and SEM results indicated that the peptides LADW and EEAEGT destroy the internal structure of starch and bind starch through intermolecular hydrogen bonds. At the same time, the ability of the peptide EEAEGT to change the structure of starch was significantly stronger than that of LADW. Results from the reducing sugar content of peptide LADW- and EEAEGT-starch complex after simulated digestion showed that the peptide EEAEGT had a stronger ability to inhibit starch hydrolysis compared with LADW (the inhibition rates of reducing sugar production were 26.87 ± 1.50 and 39.56 ± 0.82%, respectively).

In summary, this present study improves our understanding of umami peptides, promoting the practical application of tuna skeletal myosin. Furthermore, the methodology employed in this study offers novel insights for discovering hypoglycemic peptides by targeting α-glucosidase inhibition and starch hydrolysis. Moreover, through searches in the BIOPEP-UWM database and Web of Science, this study identified the peptides LADW and EEAEGT as novel α-GIPs and starch hydrolysis inhibitors.

## Figures and Tables

**Table 1:** Prediction results of physicochemical properties and potential activity of peptides.

Peptide	Molecular Weight /Da (MW)	Isoelectric Point (pI)	Net Charge	Potential Biological Activity	Umami Threshold (mg/mL)
LADW	503.55	3.77	−1	ACE inhibitor ^a^ DPP-IV inhibitor ^a^ α-glucosidase inhibitor ^a^	0.125
EEAEGT	634.24	3.47	−3	ACE inhibitor ^a^ DPP-IV inhibitor ^a^ Immunostimulating peptide ^a^ α-glucosidase inhibitor ^a^	0.125
VAEQE	574.26	3.62	−2	DPP-IV inhibitor ^a^ Allergen ^b^	0.125
MEIDD	621.23	3.39	−3	ACE inhibitor ^a^ DPP-IV inhibitor ^a^ Allergen ^b^	0.250

Note: ^a^ and ^b^ data accessed from BIOPEP database (https://biochemia.uwm.edu.pl/biopep-uwm/ (accessed on 20 July 2023)) and AllerTOPv.2.0 (http://www.ddg-pharmfac.net/AllerTOP/index.html (accessed on 20 July 2023)), respectively.

**Table 2:** ADMET prediction results of peptides LADW and EEAEGT.

Peptide	Database		Absorption			Distribution		Metabolism				
								CYP450 inhibitor				
			HIA	Caco-2	LogP	BBB	PPB	1A2	2C19	2C9	2D6	3A4
LADW	ADMETLab 2.0		0.921	−6.816	0.154	0.132	0.123	0.003	0.036	0.081	0.031	0.017
	admetSAR @ LMMD		High	Low	-	Low	-	No	No	No	No	No
	Swiss-ADME		Low	-	0.02	No	-	No	No	No	No	No
	iDrug platform		0.985	0.128 × 10^−6^	0.117	0.297	0.732	0.22	0.2	0.09	0.16	0.14
EEAEGT	ADMETLab 2.0		0.904	−7.16	−4.622	0.062	0.200	0	0.016	0.033	0	0.003
	admetSAR @ LMMD		Medium	Low	-	High	-	No	No	No	No	No
	Swiss-ADME		Low	-	-	No	-	No	No	No	No	No
	iDrug platform		0.842	1.954 × 10^−6^	−3.74	0.195	0.409	0.197	0.220	0.156	0.147	0.119
Excretion		Toxicity										
Human Clearance		Carcinogenicity		Rat Oral Acute Toxicity			DILI			Ames Toxicity		
2.074		0.145		0.173			0.042			0.005		
-		No		-			-			No		
-		-		-			-			-		
0.519		0.197		-			0.371			0.031		
1.59		0.032		0.173			0.013			0.010		
-		No		-			-			No		
-		-		-			-			-		
0.692		0.197		-			0.344			0.027		

Note: HIA, BBB, PPB, and DILI represent human intestinal absorption, blood–brain barrier, plasma–protein binding, and drug-induced liver injury, respectively. The value of Caco-2 in the ADMETLab 2.0 is greater than −5.15, indicating that it had been considered good human intestinal permeability, while the unit of Caco-2 in the iDrug platform is cm/s. The unit of human clearance is log10 of mL/min/kg [24]. LogP represents the octanol/water partition coefficient, and the smaller the Log P considered, the better the water solubility [24]. The closer the evaluation of other indicators is to 1/+, the better the potential ability.

## Data Availability

Data is contained within the article or Supplementary Materials, or are available from the corresponding author on reasonable request.

## Supplementary Materials

The following supporting information can be downloaded at: https://www.mdpi.com/article/10.3390/foods13020294/s1, Figure S1: The α-glucosidase active docking box schematic diagram; Figure S2: Molecular docking 2d diagram of acarbose and peptides (control) bound to α-glucosidase; Figure S3: MD simulation results of positive control peptide-α-glucosidases complex. Panels A to F respectively represent the RMSD, Rg, SASA, Number of H-bond, RMSF, RDF for positive control peptide-α-glucosidases complex; Figure S4: FEL, three different conformation comparisons, and the weak interaction of peptides and α-glucosidases. Panels A and B show the results of positive control peptides SVPA and GPPGPA binding to α-glucosidases, respectively. The number 1 represents the FEL and the initial (cyan), lowest energy (grey), and final (green) comparison. The number 2 represents weak interaction between peptides and surrounding residues within 5 Å of α-glucosidases. positive control peptide; Figure S5: Determination of the standard curve of standard glucose solution (2.5, 5, 10, 20, 40 mg/mL) by using the DNS method; Table S1: The electrolytes for simulated gastric fluid (SGF) and simulated intestinal juice (SIF) information list; Table S2: The 2-Hydroxy-3,5-dinitrobenzoic acid (DNS) information list (100 mL); Table S3: Results of preserve peptide sequence after digestion by gastrointestinal enzymes; Table S4: Results of hydrogen bonds and other intermolecular interaction force between peptides and α-glucosidase by molecular docking; Table S5: Results of Lennard-Jone (LJ), Coulomb interaction (Coul), and total energies of the peptides and receptors (α-glucosidase and amylose) complex in the same conditions.
