# Supplementary material for: Exploring the Blood Glucose-Lowering Potential of the Umami Peptides LADW and EEAEGT Derived from Tuna Skeletal Myosin: Perspectives from α-Glucosidase Inhibition and Starch Interaction
Source: Foods. 2024 Jan 17;13(2):294. doi: 10.3390/foods13020294 (PMC10815170; doi:10.3390/foods13020294)
Supplement: Supplementary file 1 [file foods-13-00294-s001.zip › foods-2797703-supplementary.pdf]

Exploring the Blood Glucose-Lowering Potential of the Umami Peptides LADW and EEAEGT Derived from Tuna Skeletal Myosin: Perspectives from α-Glucosidase Inhibition and Starch Interaction

Shuai Zhao [1,2,3,4], Shengbao Cai [1,2,3,4], Lixin Ding [1,2,3,4], Junjie Yi [1,2,3,4], Linyan Zhou [1,2,3,4], Zhijia Liu [1,2,3,4] and Chuanqi Chu [1,2,3,4,*]

[1] Faculty of Food Science and Engineering, Kunming University of Science and Technology, Kunming 650500, China; zhaoshuaikust1998@163.com (S.Z.); caikmust2013@163.com (S.C.); dlx2528@163.com (L.D.); junjieyi@kust.edu.cn (J.Y.); zhoulinyan916@hotmail.com (L.Z.); liuzhijia@kust.edu.cn (Z.L.)
[2] Yunnan Engineering Research Center for Fruit & Vegetable Products, Kunming 650500, China
[3] International Green Food Processing Research and Development Center of Kunming City, Kunming 650500, China
[4] Yunnan International Joint Laboratory of Green Food Processing, Kunming 650500, China
* Correspondence: chuanqichu@kmust.edu.cn; Tel.: +86-17851320636

Table S1. The electrolytes for simulated gastric fluid (SGF) and simulated intestinal juice (SIF) information list.

Reagents	Concentrations (mol/L)	Final add content	
		SGF (pH=3±0.2) (mL)	SGF (pH=8±0.2) (mL)
KCl	0.500	10.350	13.600
KH_2PO_4	0.500	1.350	1.600
$NaHCO_3$	1.000	18.750	85.000
NaCl	2.00	88.500	96.000
$MgCl_2(H_2O)_6$	0.150	0.600	2.200
$(NH_4)_2CO_3$	0.500	0.750	/

Table S2. The 2-Hydroxy-3,5-dinitrobenzoic acid (DNS) information list (100 mL).

Reagents	Add content (g)
potassium sodium tartrate	18.2
2-Hydroxy-3,5-dinitrobenzoic acid	0.63
NaOH	2.1
Phenol	0.5
Anhydrous sodium sulfite	0.5

Note: Potassium sodium tartrate was first dissolved at 45 °C, and then other reagents were added; After sitting for 7 days, DNS can be used.

Table S3. Results of preserve peptide sequence after digestion by gastrointestinal enzymes.

Server / Enzyme	BIOPEP database		PeptideCutter	
/	LADW	EEAEGT	LADW	EEAEGT
Pepsin	LADW	EEAEGT	LADW	EEAEGT
Trypsin	LADW	EEAEGT	LADW	EEAEGT
Chymotrypsin	LADW	EEAEGT	LADW	EEAEGT
Pepsin, Trypsin, and Chymotrypsin	LADW	EEAEGT	LADW	EEAEGT

Table S4. Results of hydrogen bonds and other intermolecular interaction force between peptides and α-glucosidase by molecular docking.

Peptides	Affinity energy kcal/mol	Hydrogen bonds and bond length	Van der Walls forces	alkyl interactions
LADW	-9.6	Gln279(2.4Å),Arg442(2.2Å)	Asp69,Ser157,Phe159,Arg213, Val216,Ser240,Ser241,Asp242, Phe303,Asp307,Pro312,Phe314, His351,Gln353	Tyr72,Tyr158,Phe178
EEAEGT	-8.5	Lys156(2.4Å),Ser240(2.5Å), Ser241(2.1Å),Glu277(2.7Å), Gln279(2.6,3.0Å),Arg315(2.8Å)	Ser157,Phe159,Phe178,Val216, Val232,Asp233,Gln239, Asp242, Phe303,Pro312,Leu313,Phe314, Asp352,Glu411,Arg442	-

Table S5. Results of Lennard-Jone (LJ), Coulomb interaction (Coul), and total energies of the peptides and receptors (α-glucosidase and amylose) complex in the same conditions.

Energies / System	LJ (kJ/mol)		Coul (kJ/mol)		Total energies (kJ/mol)	
	Means	Std	Means	Std	Means	Std
LADW-α-glucosidase complex	-236.53	21.91	-359.88	161.96	-596.42	183.87
EEAEGT-α-glucosidase complex	-182.59	28.48	-386.75	99.04	-569.338	127.52
LADW-amylose complex	-78.07	30.93	-60.79	37.21	-138.86	68.14
EEAEGT-amylose complex	-81.33	42.81	-95.21	57.93	-176.53	100.75

Figure S1 The α-glucosidase active docking box schematic diagram.

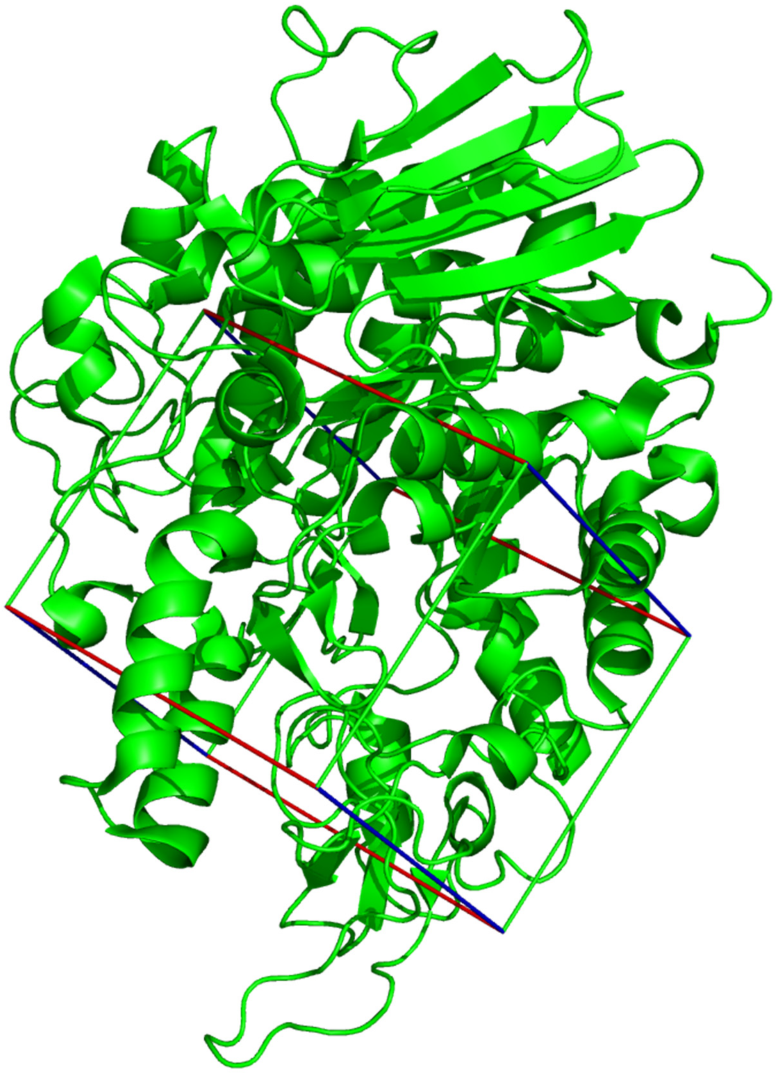

Figure S2 Molecular docking 2d diagram of acarbose and peptides (control) bound to α-glucosidase.

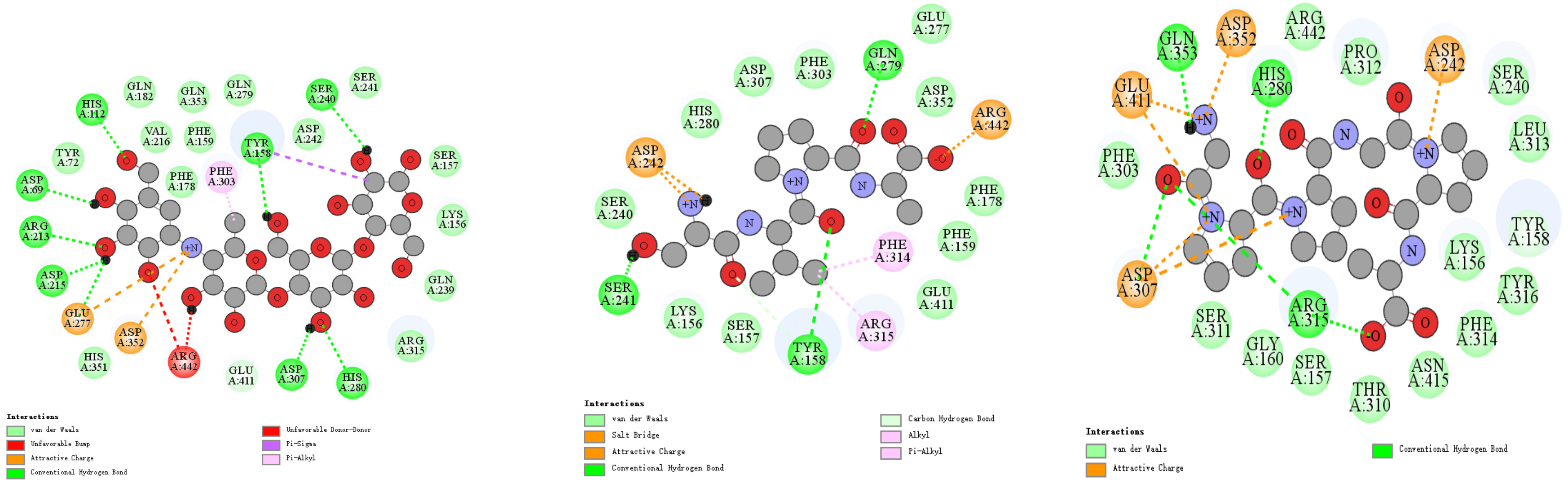

Affinity energy= -9.9 kcal/mol

Acarbose-α-glucosidase

Affinity energy= -7.8 kcal/mol

SVPA-α-glucosidase [a]

Affinity energy= -9.2 kcal/mol

GPPGPA-α-glucosidase [b]

Note: a: Peptide sequence derived from Ibrahim et al. [1]; b: peptide sequence derived from Zhou et al. [2].

Figure S3. MD simulation results of positive control peptide-α-glucosidases complex. Panels A to F respectively represent the RMSD, Rg, SASA, Number of H-bond, RMSF, RDF for positive control peptide-α-glucosidases complex.

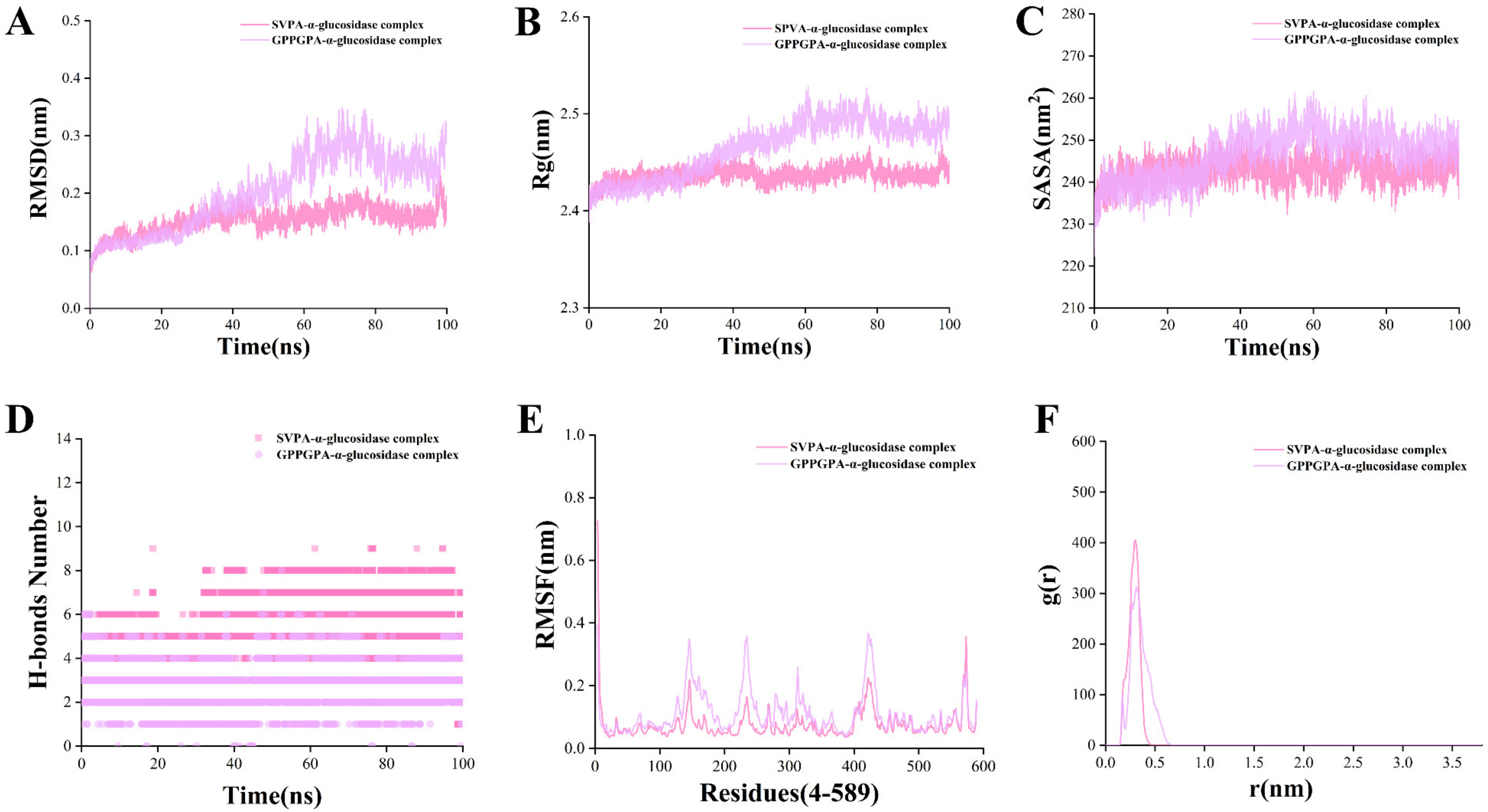

Figure S4 FEL, three different conformation comparisons, and the weak interaction of peptides and α-glucosidases. Panels A and B show the results of positive control peptides SVPA and GPPGPA binding to α-glucosidases, respectively. The number 1 represents the FEL and the initial (cyan), lowest energy (grey), and final (green) comparison. The number 2 represents weak interaction between peptides and surrounding residues within 5 Å of α-glucosidases. positive control peptide.

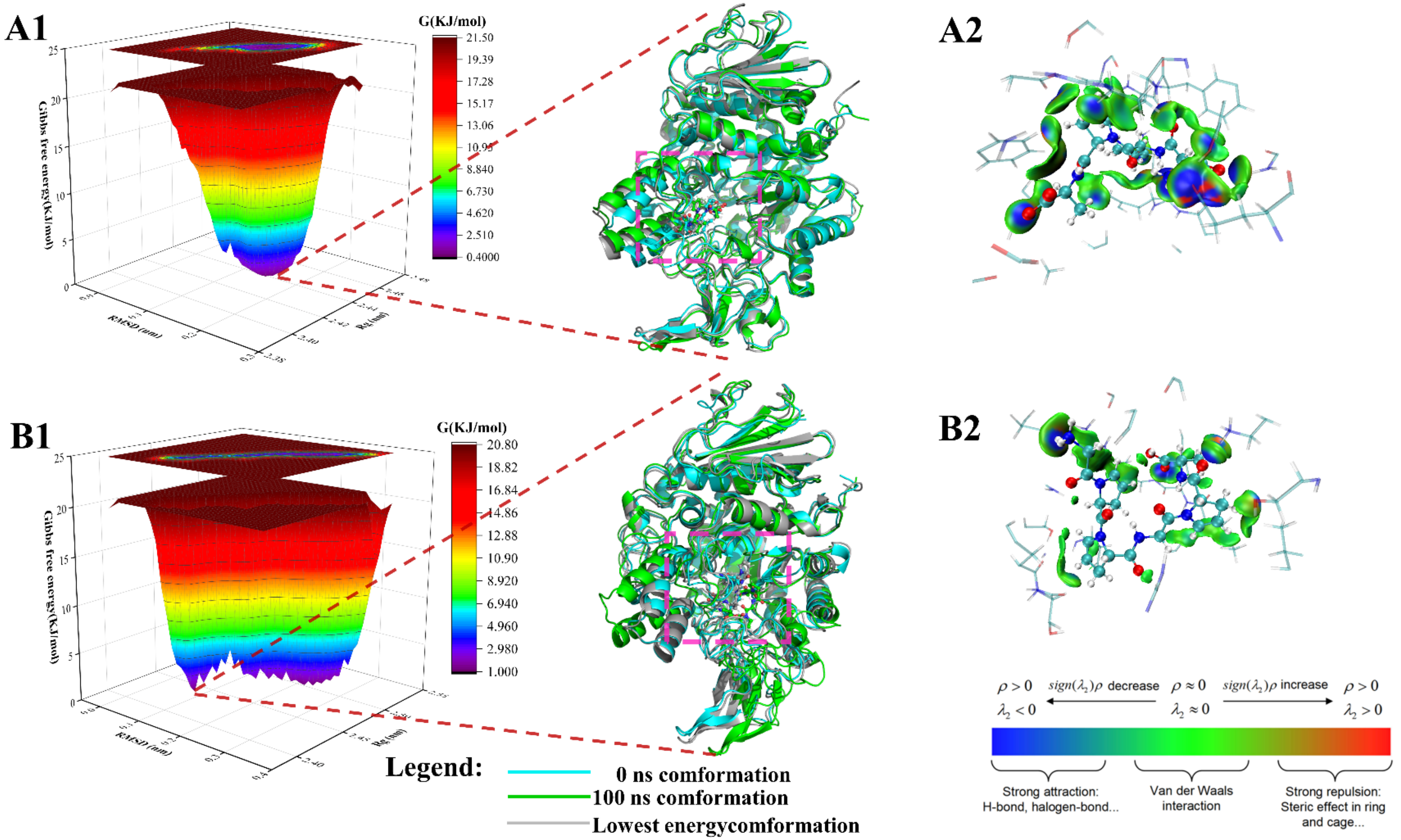

Figure S5 Determination of the standard curve of standard glucose solution (2.5, 5, 10, 20, 40 mg/mL) by using the DNS method.

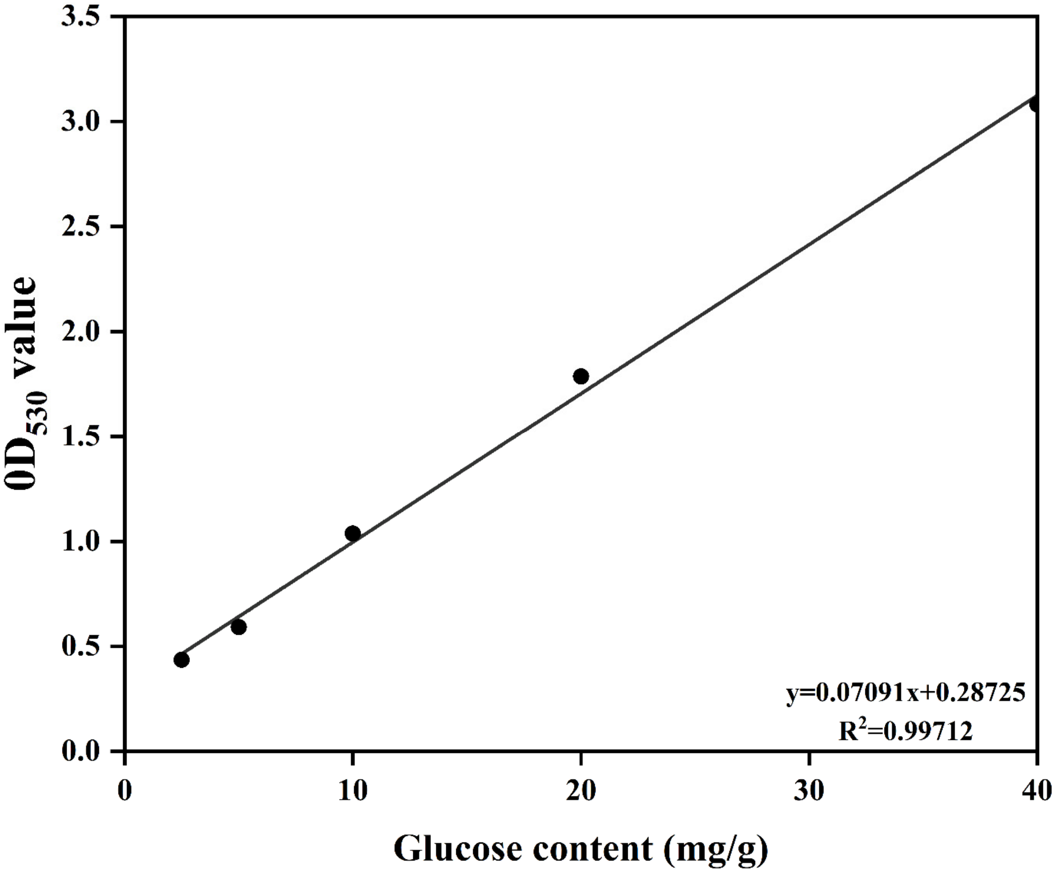

1. Ibrahim, M.A.; Bester, M.J.; Neitz, A.W.; Gaspar, A.R.M. Rational in silico design of novel α-glucosidase inhibitory peptides and in vitro evaluation of promising candidates. *Biomed. Pharmacother*. **2018**, *107*, 234–242.
2. MZhou; Ren, G.; Zhang, B.; Ma, F.; Fan, J.; Qiu, Z. Screening and identification of a novel antidiabetic peptide from collagen hydrolysates of Chinese giant salamander skin: Network pharmacology, inhibition kinetics and protection of IR-HepG2 cells. *Food Funct.* **2022**, *13*, 3329–3342.
